# Иммуногенетика первичного гиперальдостеронизма: фундаментальные исследования и их клинические перспективы

**DOI:** 10.14341/probl12783

**Published:** 2022-04-30

**Authors:** С. Х. Эристави, Н. М. Платонова, Е. А. Трошина

**Affiliations:** Национальный медицинский исследовательский центр эндокринологии; Национальный медицинский исследовательский центр эндокринологии; Национальный медицинский исследовательский центр эндокринологии

**Keywords:** первичный гиперальдостеронизм, гипертензия, аутоантитела, АТ1-рецепторы, ангиотензин II, альдостерон, альдостерон-продуцирующая аденома, двусторонний гиперальдостеронизм

## Abstract

Первичный гиперальдостеронизм (ПГА) — самая частая форма эндокринной гипертензии. Причиной развития данного состояния до недавнего времени считалось наличие генетических мутаций, однако множество исследований декларирует, что заболевание может быть полиэтиологично, следствием как генетических мутаций, так и аутоиммунных триггеров и клеточных кластеров альдостерон-продуцирующих клеток, диффузно расположенных в надпочечнике, и в клубочковой, и в пучковой зонах, а также непосредственно под капсулой надпочечника. В недавнее время было описано действие аутоантител к рецепторам 1-го типа ангиотензина II у пациентов с отторжением почечного трансплантата, при преэклампсии и при ПГА. Диагностическая роль антител при обеих формах ПГА (альдостерон-продуцирующей аденоме и двустороннем гиперальдостеронизме) требует уточнения. Диагностика и подтверждение очага гиперсекреции альдостерона — многоэтапная процедура, требующая длительных временных и экономических затрат. Актуальность своевременной диагностики ПГА заключается в снижении медико-социальных потерь. В данной работе суммированы знания о генетических мутациях и представлены все оригинальные исследования, посвященные аутоантителам при ПГА, а также обсуждены диагностические возможности и ограничения имеющихся методов первичной и дифференциальной диагностики заболевания и перспективы терапии.

## Abstract (en)

Primary hyperaldosteronism (PHA) is the most common form of endocrine hypertension. Until recently, the reason for the development of this condition was believed to be the presence of genetic mutations, however, many studies declare that the disease can be polyetiologic, be the result of genetic mutations and autoimmune triggers or cell clusters of aldosterone-producing cells diffusely located in the adrenal gland at the zona glonerulosa, zona fasculata, zona reticularis, as well as directly under the adrenal capsule. Recently, the actions of autoantibodies to type 1 angiotensin II receptors have been described in patients with renal transplant rejection, with preeclampsia, and with primary hyperaldosteronism. The diagnostic role of antibodies in both forms of PHA (aldosterone-producing adenoma and bilateral hyperaldosteronism) requires clarification. Diagnosis and confirmation of the focus of aldosterone hypersecretion is a multi-stage procedure that requires a long time and economic costs. The relevance of timely diagnosis of primary hyperaldosteronism is to reduce medical and social losses. This work summarizes the knowledge about genetic mutations and presents all the original studies devoted to autoantibodies in PHA, as well as discusses the diagnostic capabilities and limitations of the available methods of primary and differential diagnosis of the disease and the prospects for therapy.

Артериальная гипертензия — распространенное состояние, встречается у 20% населения в развитых странах [1], выделяют первичную (или эссенциальную) гипертонию, которая характерна для первичных поражений сердечно-сосудистой системы, и вторичную, при которой поражаются органы, участвующие в системе регуляции артериального давления.

Наиболее частая причина вторичной гипертонии — это первичный гиперальдостеронизм (ПГА). ПГА встречается в 5–10% случаев у пациентов с гипертонической болезнью и в 20% случаев у пациентов с гипертензией, резистентной к терапии [2][3].

ПГА впервые был описан в 1955 г. Джереми Конном [4].

ПГА характеризуется избыточной секрецией альдостерона. Альдостерон — минералокортикоидный гормон, который регулирует кровяное давление, воздействуя на водно-натриевый баланс. Альдостерон секретируется в клубочковой зоне коры надпочечников под влиянием двух главных стимуляторов: ангиотензина II и калия [5]. Ангиотензин II связывается с рецепторами 1-го типа ангиотензина II на мембране клетки, которые расположены в трофобластах, гладкой мускулатуре и эндотелии сосудов, а также в экстрагломерулярных мезаганглиях в почках [6]. Далее происходит активация фосфолипазы С, которая активируется субъединицами Gαq или Gβγ G-белка, происходит каскад реакций, в результате которых мембраны клеток деполяризуются, открываются кальциевые каналы и кальций активно поступает в клетки. Повышение внутриклеточного кальция влияет на экспрессию гена CYP11B2 (альдостеронсинтетаза) и синтез альдостерона [6].

## ЭТИОЛОГИЧЕСКИЕ ПРИЧИНЫ РАЗВИТИЯ ПЕРВИЧНОГО ГИПЕРАЛЬДОСТЕРОНИЗМА

Избыточная секреция альдостерона может быть вызвана герминальными и соматическими мутациями генов, кодирующих ионные каналы в клубочковой зоне коры надпочечников, ведущих к развитию как одностороннего и двустороннего гиперальдостеронизма в рамках семейных форм, так и к развитию альдостерон-продуцирующих кластеров [7–9], а также под влиянием иммуногенетических триггеров [2]. В данной статье мы хотим обсудить патогенетическую и клиническую роль аутоантител к рецепторам 1 типа альдостерона 2, а также суммировать знания о генетических мутациях и возможных подходах к диагностике и терапии.

## Генетические мутации

Причины развития ПГА до сих пор изучаются, однако уже давно известно, что главенствующую патогенетическую роль имеют генетические мутации ионных каналов, которые приводят к увеличению концентрации внутриклеточного кальция и гиперсекреции альдостерона. Считается, что в более чем 50% случаев причиной одностороннего ПГА является соматическая мутация генов, кодирующих ионные каналы, участвующие в ренин-ангиотензин-альдостероновой системе [10].

Основные мутации при одностороннем ПГА: KCNJ5, CACNA1D, ATP1A1, ATP2B3.

Все мутации ведут к избыточной секреции альдостерона, длительной стимуляции клубочковой зоны коры надпочечников и развитию гиперплазии, а впоследствии — аденомы надпочечника.

Мутация гена KCNJ5 встречается у 38% пациентов с односторонним ПГА [11], приводит к потере селективности K-канала и избыточному поступлению ионов Na в клетки, деполяризации мембраны, открытию кальциевых каналов, стимуляции CYP11B2 и, как следствие, избыточной секреции альдостерона.

Данная мутация чаще встречается у людей азиатской расы, молодых женщин и характеризуется низким содержанием калия в крови и большими размерами аденомы [12].

Вторая по распространенности мутация гена CACNA1D характеризуется нарушением работы потенциалзависимых кальциевых каналов, которые реагируют на деполяризацию мембраны, однако в случае данной мутации эта связь нарушается и кальциевые каналы пропускают кальций внутрь клетки постоянно, что ведет к стимуляции CYP11B2 и избыточной секреции альдостерона.

Также встречаются мутации генов ATP1A1 и АTP2B3, которые кодируют Na++/K+-АТФ-азу и Cа++-АТФ-азу соответственно. При первой происходят деполяризация мембраны, открытие потенциалзависимых кальциевых каналов, стимуляция CYP11B2 и избыточная секреция альдостерона. При второй нарушается селективность кальциевых каналов [13].

Также были идентифицированы транскрипты, кодирующие другие ферменты, участвующие в метаболизме стероидов, а именно HSD3B2 (кодирующий 3-β-гидроксистероиддегидрогеназу II типа) и CYP21 (кодирующий 21-гидроксилазу). Последний ген также был сверхэкспрессирован в альдостерон-продуцирующих аденомах в последовательном анализе экспрессии генов (SAGE) и подтвержден секвенированием методом ISH (in situ hybridization).

Ген рецептора, связанный с G-белком, рецептор серотонина 4 (рецептор гидрокситриптамина 4, HTR4), который стимулирует высвобождение циклического аденозинмонофосфата в ответ на серотонин, был идентифицирован при альдостероме. Аберрантная экспрессия рецепторов, связанных с G-белком, таких как HTR4, среди прочего, была предложена в качестве предполагаемого механизма дерегуляции продукции стероидов при альдостероме.

При семейных формах ПГА отмечены следующие мутации: при первом типе происходит неравный кроссинговер между CYP11B1 (кодирует 11β-гидроксилазу) и CYP11B (кодирует синтез альдостерона) на хромосоме 8q24, что ведет к глюкокортикоидзависимому альдостеронизму [14]. Второй тип семейного ПГА ассоциирован с мутацией гена CLCN2. Оба типа проявляются как односторонним, так и двусторонним гиперальдостеронизмом [15]. При обеих вариациях 3-го типа выявлена мутация в гене GIRK4 (кодируемом KCNJ5), что приводит к низкой концентрации калия внутри клетки, избыточному поступлению натрия, деполяризации мембраны и гиперсекреции альдостерона по указанному выше пути. Чаще всего данный тип проявляется двусторонним гиперальдостеронизмом, однако клинические проявления обеих вариаций 3 типа различаются, тип А проявляется ювенильным тяжелым гиперальдостеронизмом, тип Б — мягким течением гиперальдостеронизма [10][15]. При 4-м типе определена мутация CACNA1H, морфологически проявляется как односторонней, так и двусторонней гиперплазией надпочечников, ювенильным тяжелым гиперальдостеронизмом и нарушением умственного развития [15]. Пятый тип ассоциирован с мутацией CACNA1D, морфологически надпочечники остаются неизменными, однако проявляется ювенильным ПГА, судорогами и неврологическими осложнениями. Две последние мутации проявляются нарушением селективности кальциевых каналов [15].

## Кластеры альдостеронпродуцирующих клеток

Как было сказано выше, альдостерон продуцируется в клубочковой зоне коры надпочечников, и точка приложения вышеперечисленных мутаций, соответственно, тоже находится в клубочковой зоне. Однако в 2010 г. были описаны кластеры альдостеронпродуцирующих клеток, диффузно расположенных в надпочечнике, и в клубочковой и в пучковой зонах, а также непосредственно под капсулой надпочечника [7][8]. Их количество увеличивается с возрастом, они локализуются в обоих надпочечниках. Вероятно, данные кластеры являются предшественниками альдостером, так как обе нозологии ассоциированы с одними мутациями (в генах CACNA1D, ATP1A1 и ATP2B3) [9].

## Аутоантитела к рецепторам 1-го типа ангиотензина II

В последние годы появляется все больше данных о потенциальном влиянии аутоиммунных триггеров на рецепторы 1-го типа к ангиотензину II, которые стимулируют секрецию альдостерона по указанному выше пути при одностороннем и двустороннем ПГА [2][3][4][16–18]. Однако ввиду небольшого количества клинических исследований, проведенных в данной области, определение антител к АТ1-рецепторам до сих пор не вошло в рутинную практику. Циркулирующие антитела к рецепторам 1-го типа ангиотензина II впервые были определены в плазме крови пациентов с ПГА учеными из Италии G. Rossitto et al. Ими было выявлено, что у 92% пациентов с альдостеронпродуцирующими аденомами в 2 раза повышен титр антител в сыворотке крови по сравнению с титром антител у пациентов с идиопатическим ПГА [2]. Впоследствии было доказано, что антитела к рецепторам 1-го типа ангиотензина II обладают агонистическим действием и стимулируют секрецию альдостерона, а также обладают слабым вазоконстрикторным действием. Также было замечено, что при наличии низких концентраций альдостерона в сыворотке антитела к рецепторам 1-го типа ангиотензина II изменяют аллостерическую конфигурацию рецепторов 1-го типа ангиотензина II, тем самым улучшая связывание и усиливая эффект низких концентраций альдостерона [17]. Последующее исследование Y. Li et al. продемонстрировало преобладание антител у пациентов с двусторонним ПГА по сравнению с пациентами с альдостеромой (75% против 46%). Преобладание антител у пациентов с двусторонним ПГА прямо противоречит исследованию G. Rossitto et al. [16]. Такие выраженные различия в результатах данных исследований объясняются тем, что авторы использовали разные методы исследования. G. Rossitto применял иммуноферментный анализ для определения уровня антител к АТ1-рецепторам, тогда как D. Kem и Y. Li наблюдали экспрессию альдостерона путем стимуляции клеток клубочковой зоны антителами (т.е. использовали иммуногистохимическое исследование). T. Williams объясняет столь разные результаты тем, что у пациентов с двусторонним и односторонним ПГА различные фенотипы чувствительности рецепторов к ангиотензину II, а следовательно, и к антителам [19]. К тому же пациенты с двусторонним ПГА более чувствительны к действию ангиотензина, а также у них определяются более высокие показатели альдостерона в плазме крови в утреннее время в положении лежа, чем у пациентов с АПА [20][21].

A. Mottl et al. описывают 5 различных структурных вариаций рецепторов 1-го типа к ангиотензину II [22], помимо основных функций, есть еще и другие функции данных рецепторов, что подтверждает их полиморфизм [23].

Результаты систематического обзора показывают, что доступная в настоящее время литература слишком разнородна, чтобы делать значимые выводы (табл. 1).

**Table:** Таблица 1. Результаты исследованийTable 1. Research results

Заболевание	Количество исследованных образцов	Преобладание антител к АТ1-рецептору (100%)	Исследование
Первичный гиперальдостеронизм	
АПА+ИПА	13	31	D. Kem [13]
АПА	26	92	G. Rossitto [7]
АПА	13	46	Y. Li [12]
ИПА	12	75	Y. Li [12]
АПА	15	23	C. Sabbadin et al. [8]
ИПА	29	34	C. Sabbadin [8]
Преэклампсия	25	100	G. Wallukat et al. [21]
16	100	T. Thway et al. [22]
5 и 19	80 и 89	T. Walther et al. [23]
31	81	X. Yang et al. [24]
30	70	F. Herse et al. [25]
37	95	A. Siddiqui et al. [26]
58	48	S. Zhang et al. [27]
Отторжение почечного трансплантата и злокачественная гипертензия	16	100	D. Dragun [16]

Очевидно одно — циркуляция антител к рецепторам может длительно стимулировать клетки клубочковой зоны, в результате чего повышается риск развития гиперпролиферативных состояний и соматических мутаций, которые впоследствии могут стать причиной развития альдостеронпродуцирующих аденом. Был исследован уровень антител к рецепторам 1-го типа ангиотензина II у пациентов с односторонним ПГА до и спустя 1 мес после адреналэктомии, значительного снижения уровня антител выявлено не было, что позволяет утверждать, что источник секреции антител расположен не в надпочечниках [24].

Данная информация может изменить взгляд на патогенез ПГА, который раньше не рассматривался как аутоиммунное заболевание. Любопытно, но в эндокринологии и кардиологии увеличивается количество аутоиммунных заболеваний, проявляющихся секрецией аутоантител к G-связанным рецепторам. Известны такие примеры, как болезнь Грейвса, при которой наблюдается стимуляция антител к рецепторам тиреотропного гормона, также в патогенезе дилатационной кардиомиопатии антитела стимулируют β1-адренергические рецепторы, и при различных формах эссенциальной гипертонии происходит стимуляция α1-адренергических рецепторов [19]. При анализе литературы обращает на себя внимание тот факт, что аутоантитела к рецепторам 1-го типа ангиотензина II определяются у двух групп населения: у женщин с преэклампсией и у пациентов с отторжением почечного трансплантата [20][21][25–30]. Антитела преимущественно преобладают у пациенток с преэклампсией, определяются в 18 нед, и их количество увеличивается со сроком беременности, значительно снижаются после родоразрешения, но продолжают циркулировать в крови вплоть до года после родов [31]. Отмечено, что уровень антител и тяжесть преэклампсии прямо коррелируют [32].

Давно известно, что антитела к рецепторам 1-го типа ангиотензина II определяются и у пациентов с почечными аллотрансплантантами. Маркером высокого риска отторжения почечного трансплантата является наличие антител к HLA (human leukocyte antigens — человеческому лейкоцитарному антигену), однако в 2005 г. D. Dragun отметил отторжение трансплантата у 16 пациентов без антител к HLA, но с наличием антител к рецепторам 1-го типа ангиотензина II [25]. Позже было доказано, что антитела к рецепторам 1-го типа ангиотензина II вызывают отторжение почечного трансплантата, а их титр может коррелировать с риском отторжения трансплантата в позднем послеоперационном периоде [33][34].

## ДИАГНОСТИКА

Несмотря на различия в этиологии, все формы ПГА диагностируются по одному стандарту.

Это многоступенчатый процесс, который до сих пор вызывает определенные трудности в непрофильных медицинских учреждениях [2][35].

Симптомы ПГА неспецифические — это стойкая, резистентная к терапии артериальная гипертензия и, при определенных формах, гипокалиемия, проявляющаяся нарушениями сердечного ритма, ослаблением скелетных мышц, а также неврологическими нарушениями.

Несмотря на то что имеющиеся методы инструментальной и лабораторной диагностики отлично справляются с поставленными задачами, они имеют определенные недостатки, такие как высокая вариабельность результатов на преаналитическом этапе (на этапе забора крови), зависимость результатов от внешних условий (лекарственных препаратов, возраста, диеты лабораторных условий).

В настоящее время золотым стандартом дифференциальной диагностики является сравнительный селективный забор крови из надпочечниковых вен [35], который, несмотря на все преимущества, сопряжен с определенными недостатками — дефицит специалистов, обладающих техникой выполнения манипуляции, оборудованных рентгеноперационных, дороговизна исследования, риск развития интраи послеоперационных осложнений и необходимость госпитализации пациента в стационар [36][37]. Неинвазивной альтернативой проведения сравнительного селективного забора крови из надпочечниковых вен является проведение позитронно-эмиссионной томографии с 11С-метомидатом. Метомидат — это лиганд с одинаковым сродством к ферментам CYP11B1 и CYP11B2, что ограничивает использование метода, так как экспрессия фермента CYP11B1 встречается и при глюкокортикоидсекретирующих аденомах, и при ПГА [38][39]. Также применяют сцинтиграфию с 123-йодметомидатом для визуализации гормонально-активных образований в надпочечниках. Однако данные методы обладают низкой специфичностью в диагностике ПГА. Позитронно-эмиссионная томография с 11С-метомидатом обладает 76% чувствительностью и 87% специфичностью в диагностике ПГА [40].

Проведение генетического типирования и ядерных методов диагностики возможно в условиях неэффективности сравнительного селективного забора крови как альтернативных методов диагностики, с целью выбора дальнейшей тактики лечения. Обращает на себя внимание использование секвенирования нового поколения как с целью диагностики генетических мутаций, встречающихся при ПГА, так и с целью поиска новых генов-кандидатов, потенциально ведущих к развитию альдостеронпродуцирующих аденом и наследственных форм ПГА [23]. Перспективным методом диагностики является определение аутоантител к рецепторам 1-го типа ангиотензина II.

Своевременная диагностика и лечение имеют крайне важное значение как в медико-социальном, так и в экономическом плане. ПГА характеризуется высокой частотой сердечно-сосудистых заболеваний и летальных исходов [41]. Своевременная диагностика данного заболевания позволит вовремя начать адекватное лечение, предотвратить развитие поздних осложнений и летальных исходов.

## ПЕРСПЕКТИВЫ В ЛЕЧЕНИИ

Методом выбора в лечении ПГА с односторонней гиперпродукцией альдостерона (альдостеронпродуцирующая аденома и односторонняя надпочечниковая гиперплазия) является лапароскопическая адреналэктомия. Ввиду того, что кластеры с альдостеронпродуцирующими клетками могут быть причиной рецидива в контралатеральном надпочечнике, необходим динамический послеоперационный контроль при известных мутациях. Терапией выбора при двусторонней гиперпродукции альдостерона остается длительное назначение антагонистов минералокортикоидных рецепторов. В настоящее время ведутся исследования по изучению эффективности и безопасности новых лекарственных препаратов — нестероидных антагонистов минералокортикоидных рецепторов, которые обладают более высокой рецепторной чувствительностью и более сильным связыванием с рецепторами по сравнению с доступной на данный момент терапией антагонистами минералокортикоидных рецепторов (спиронолактон, эплеренон) [42].

## ОБСУЖДЕНИЕ

ПГА — медико-социально значимое заболевание, своевременная диагностика и лечение которого позволяют улучшить качество жизни, предупредить летальные исходы и нивелировать экономические затраты на лечение осложнений, таких как сердечно-сосудистые и неврологические поражения. ПГА не обладает специфическими симптомами, которые могут привести пациента к эндокринологу, поэтому важны настороженность и осведомленность врачей смежных специальностей. Лабораторно-инструментальная диагностика направлена на следствие заболевания — определение повышенного уровня альдостерона, низкого уровня ренина и калия, что, безусловно, ограничивает ее использование в определенных случаях. В мировом научном сообществе обсуждаются новые методы диагностики, направленные на первопричину, которая может быть представлена генетическими мутациями или аутоиммунными реакциями. Представлены новые методы межнозологической диагностики ПГА, которые можно использовать наряду с генетическим секвенированием при неэффективности сравнительного селективного забора крови из надпочечниковых вен.

Обращает на себя внимание то, что благодаря новым методам генетической диагностики, а именно генного секвенирования нового поколения, границы в межнозологической классификации ПГА стираются и встает вопрос о том, являются ли кластеры альдостеронпродуцирующих клеток переходной формой между двусторонним и односторонним ПГА? Провести межнозологическую диагностику позволит определение мутаций в генах: KCNJ5, CACNA1D, ATP1A1 и ATP2B3, данные мутации встречаются при одностороннем ПГА и альдостеронпродуцирующих кластерах, выявить наличие альдостеронпродуцирующих клеточных кластеров возможно благодаря проведению иммуногистохимии, после проведенного оперативного вмешательства. Необходима настороженность при наличии альдостеронпродуцирующих клеточных кластеров, так как данное состояние может быть двусторонним и стать причиной рецидива ПГА в контралатеральном надпочечнике. Можно ли выделить промежуточные формы ПГА? Почему возникают рецидивы в контралатеральных надпочечниках после односторонних адреналэктомий? Какую терапию мы можем предложить пациентам с подтвержденными генетическими мутациями? Перспективным методом таргетной генной терапии является применение системы CRISPR/Cas9. Практически все мутации опосредованно или непосредственно влияют на кальциевые каналы, что увеличивает количество внутриклеточного кальция. Вероятно, именно кальциевые каналы являются потенциальной мишенью в терапии ПГА. Однако ограничительными рамками служит то, что кальциевые каналы имеют витальное значение в физиологии человека, и неприцельное воздействие на них может повлечь за собой развитие жизнеугрожающих состояний.

Патогенетическая и клиническая роль аутоантител к АТ1-рецепторам при ПГА малоизучена, однако очевидно, что данная информация может быть недостающим звеном в общей картине диагностики и лечения заболевания. В связи с тем что доступная на данный момент литература разнородна и проведенные исследования в данной области не были проведены по одному стандарту, для более точного определения роли антител и их количественной оценки необходимо провести исследования с большим количеством участников и одновременным применением обоих диагностических методов (иммуноферментный анализ и иммуногистохимические исследования).

Уровень антител к рецепторам 1-го типа ангиотензина II при соматических мутациях, подтвержденных у пациентов с односторонними формами ПГА, не исследовался, однако данная информация может быть полезна в понимании взаимосвязи соматических мутаций и титра антител при ПГА и позволит ответить на множество незакрытых вопросов в патогенезе заболевания.

## ЗАКЛЮЧЕНИЕ

ПГА — это заболевание, требующее междисциплинарной настороженности, ведь своевременная терапия снижает медико-социальные и экономические потери.

Более глубокое понимание патогенетических механизмов ПГА может привести к выявлению надежных диагностических и прогностических биомаркеров, а также к более чувствительным и специфичным методам скрининга, позволит рекомендовать новые терапевтические подходы к заболеванию. Для этого необходимо проведение фундаментальных и клинических исследований, которые позволят открыть новые горизонты в понимании заболевания.

В настоящий момент в ФГБУ «НМИЦ эндокринологии» Минздрава России разрабатывается проект по изучению роли аутоантител к рецепторам 1-го типа ангиотензина II при ПГА.

## ДОПОЛНИТЕЛЬНАЯ ИНФОРМАЦИЯ

Источник финансирования. Работа выполнена по инициативе авторов без привлечения финансирования.

Конфликт интересов. Авторы декларируют отсутствие явных и потенциальных конфликтов интересов, связанных с публикацией настоящей статьи.

Участие авторов. Эристави С.Х. — поиск и обработка материалов, анализ полученных данных, написание текста, участие в подготовке публикаций; Платонова Н.М. — формулирование научной проблемы, анализ полученных данных, участие в подготовке публикаций; Трошина Е.А. — формулирование научной проблемы, анализ полученных данных. Все авторы одобрили финальную версию статьи перед публикацией, выразили согласие нести ответственность за все аспекты работы, подразумевающую надлежащее изучение и решение вопросов, связанных с точностью или добросовестностью любой части работы.
